# Supplementary material for: Vascular smooth muscle RhoA counteracts abdominal aortic aneurysm formation by modulating MAP4K4 activity
Source: Commun Biol. 2022 Oct 7;5:1071. doi: 10.1038/s42003-022-04042-z (PMC9546906; doi:10.1038/s42003-022-04042-z)
Supplement: Supplementary file 2 — Supplementary information [file 42003_2022_4042_MOESM2_ESM.pdf]

Supplementary Information

Vascular smooth muscle RhoA counteracts abdominal aortic aneurysm formation by modulating MAP4K4 activity

Md Rasel Molla, Akio Shimizu, Masahiro Komeno, Nor Idayu A. Rahman, Joanne Ern Chi Soh, Le Kim Chi Nguyen, Mahbubur Rahman Khan, Wondwossen Wale Tesega, Si Chen, Xiaoling Pang, Miki Tanaka-Okamoto, Noriyuki Takashima, Akira Sato, Tomoaki Suzuki, Hisakazu Ogita

Supplementary Tables 1–3

Supplementary Figures 1–14

Supplementary Table 1 Clinical characteristics of patients analyzed in this study.

Total (n)	30
Age (year)	72.7±1.7
Male/Female (n)	25/5
BMI (kg/m^2)	23.0±0.7
Heart rate (bpm)	77±3
Systolic BP (mmHg)	128±3
Red blood cell (×10^4/μL)	427±10
ALT (IU/L)	18±2
LDL (mg/dL)	112±6
HbA1c	6.0±0.1

BMI: body mass index, BP: blood pressure, ALT: alanine aminotransferase,

LDL: low density lipoprotein cholesterol, Hb: hemoglobin

Supplementary Table 2 Information of the primary antibodies used in this study.

Name (Clone No.)	Source	Type	Company	Cat. No.	Dilution ratio
RhoA (67B9)	Rabbit	Monoclonal	Cell Signaling Technology	2117	WB (1:1000), IF and IHC (1:200)
RhoB (C-5)	Mouse	Monoclonal	Santa Cruz Biotechnology	sc-8084	IF (1:200)
GAPDH (3H12)	Mouse	Monoclonal	Medical & Biological Laboratories	M171-3	WB (1:1000)
α-Actin (1A4)	Mouse	Monoclonal	Santa Cruz Biotechnology	sc-32251	WB (1:1000), IF (1:200)
α-MHC (MF20)	Mouse	Monoclonal	R&D Systems	MAB4470	IF (1:500)
Aggrecan (4F4)	Mouse	Monoclonal	Santa Cruz Biotechnology	sc-33695	IF (1:200)
CD31 (SZ31)	Rat	Monoclonal	Dianova	DIA-310	IF (1:200)
CD31	Rabbit	Polyclonal	Abcam	ab28364	WB (1:1000)
P-p38 MAPK [Thr180/Tyr182] (D3F9)	Rabbit	Monoclonal	Cell Signaling Technology	4511	WB (1:1000), IF and IHC (1:200)
p38 MAPK (D13E1)	Rabbit	Monoclonal	Cell Signaling Technology	8690	WB (1:1000)
P-p44/42 MAPK [Thr202/Tyr204] (D13.14.4E)	Rabbit	Monoclonal	Cell Signaling Technology	4370	WB (1:1000), IF and IHC (1:200)
p44/42 MAPK (137F5)	Rabbit	Monoclonal	Cell Signaling Technology	4695	WB (1:1000)
P-MAP4K4	Rabbit	Polyclonal	Bioss	bs-5491R	WB (1:1000), IF and IHC (1:200)
MAP4K4	Rabbit	Polyclonal	Proteintech	55247-1-AP	WB (1:1000), IP (1:100)
CD68	Rabbit	Polyclonal	Abcam	ab125212	IF (1:200)
F4/80 (C-7)	Mouse	Monoclonal	Santa Cruz Biotechnology	sc-377009	IF (1:200)
MMP2 (8B4)	Mouse	Monoclonal	Santa Cruz Biotechnology	sc-13595	IF (1:200)
MMP9 (7-11C)	Mouse	Monoclonal	Santa Cruz Biotechnology	sc-13520	IF (1:200)
TIMP1 (G-6)	Mouse	Monoclonal	Santa Cruz Biotechnology	sc-365905	IF (1:200)
TIMP2 (3A4)	Mouse	Monoclonal	Santa Cruz Biotechnology	sc-21735	IF (1:200)
GFP (mFX75)	Mouse	Monoclonal	Wako Pure Chemical Industries	012-22541	WB (1:1000)
GFP (mFX73)	Mouse	Monoclonal	Wako Pure Chemical Industries	012-20461	IP (1:100)
P-MLC2 [Thr18/Ser19]	Rabbit	Polyclonal	Cell Signaling Technology	3674	WB (1:1000)
MLC2 (E-4)	Mouse	Monoclonal	Santa Cruz Biotechnology	sc-28329	WB (1:1000)
Phospho-MYLK [Ser1760]	Rabbit	Polyclonal	Invitrogen	44-1085G	WB (1:1000)
MYLK (A-8)	Mouse	Monoclonal	Santa Cruz Biotechnology	sc-365352	WB (1:1000), IP (1:100)
PP2A-Cα/β (1D6)	Mouse	Monoclonal	Santa Cruz Biotechnology	sc-80665	WB (1:1000), IP (1:100), IF (1:200)
Set	Rabbit	Polyclonal	Novusbio	NBP1-33713	WB (1:2000), IF (1:200)
HA	Rabbit	Polyclonal	Medical & Biological Laboratories	561	WB (1:1000)
Vimentin (D21H3)	Rabbit	Monoclonal	Cell Signaling Technology	5741	WB (1:1000)

IF: immunofluorescence, IHC: immunohistochemistry, IP: immunoprecipitation, WB: western blotting

Supplementary Table 3 List of primer sets used in qPCR of this study.

Human genes	Forward primer (5'-3')	Reverse primer (5'-3')
RHOA	AGCCTGTGGAAAGACATGCTT	TCAAACACTGTGGGCACATAC
GAPDH	AGCCACATCGCTCAGACAC	GCCCAATACGACCAAATCC

Mouse genes	Forward primer (5'-3')	Reverse primer (5'-3')
Gapdh	CCAAAAGGGTCATCATCTCC	GTCATGAGCCCTTCCACAAT
Eln	CGGGTCTGACAGCGGTAGT	CTCCAAGTCCTCCAGGACCT
Fbn1	GGACGCCAATTTGGAGGCT	CTTTCAGCGCATCGTGTCCT
Col1a1	GTCTGGTTTGGAGAGAGCAT	CAGTGATAGGTGATGTTCTGGG
Acta2	GTCCCAGACATCAGGGAGTAA	TCGGATACTTCAGCGTCAGGA
Mylk	TGGGGGACGTGAAACTGTTTG	GGGGCAGAATGAAAGCTGG
Myh11	GAGCAAACTCAGGAGAGGAAAC	GTCCCGAGCGTCCATTTCTTC
Il1b	GAAATGCCACCTTTTGACAGTG	TGGATGCTCTCATCAGGACAG
Il6	CTCTGGGAAATCGTGGAAAT	CCAGTTTGGTAGCATCCATC
Tnf	CCCTCACACTCAGATCATCTTCT	GCTACGACGTGGGCTACAG
Ccl2	GCCCCACTCACCTGCTGCTACT	CCTGCTGCTGGTCATCCTCTTGT
Mmp2	AGAGAGAGGCCCTCAGTTGCT	ACTCCAGTTAAAGGCAGCATCTAC
Mmp9	CTTCTGGCGTGTGAGTTTCCA	ACTGCACGGTTGAAGCAAAGA
Timp1	GCAACTCGGACCTGGTCATAA	CGGCCCGTGATGAGAAACT
Timp2	TCAGAGCCAAAGCAGTGAGC	GCCGTGTAGATAAACTCGATGTC

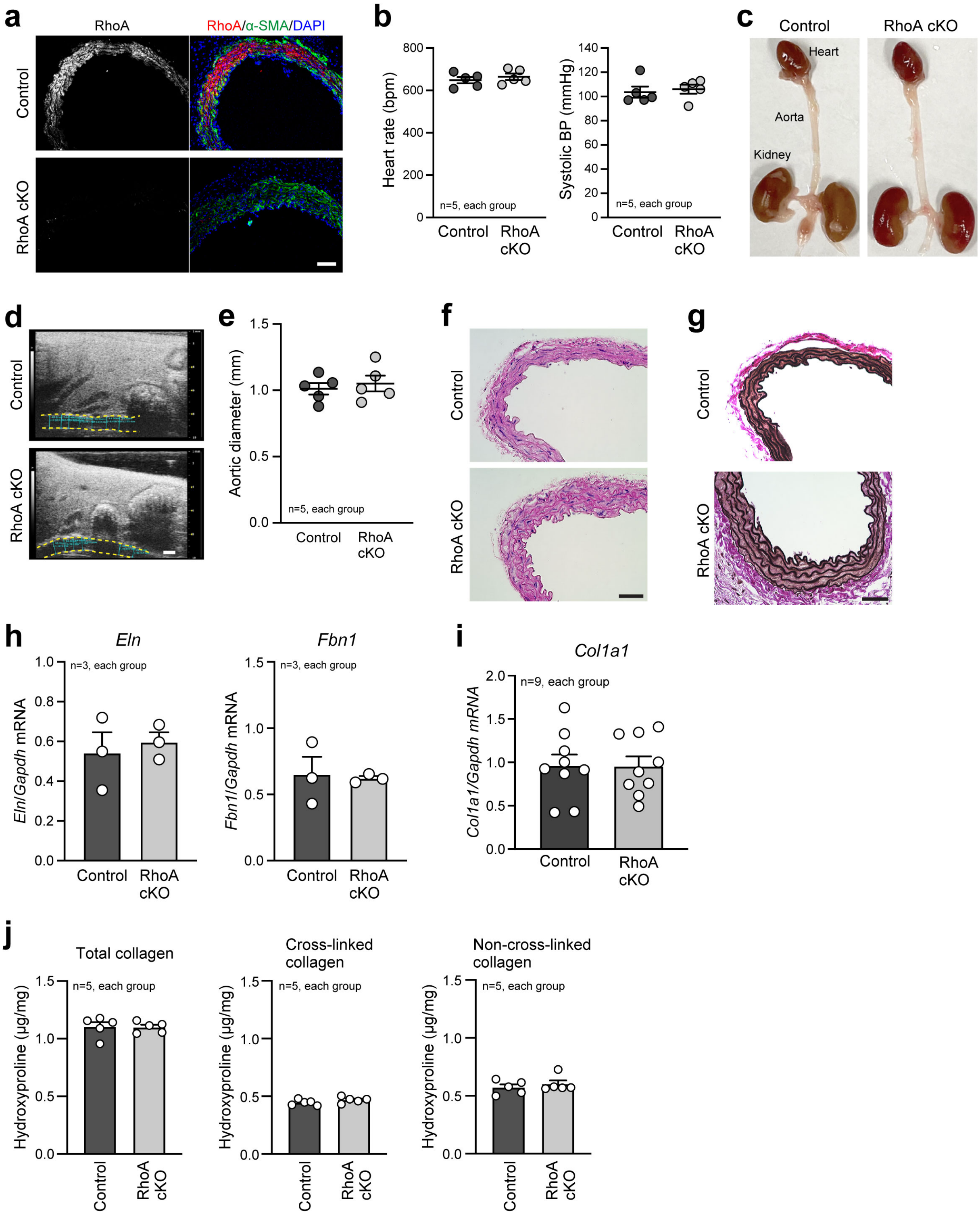

Supplementary Fig. 1 Basal characteristics of the aorta in control and RhoA cKO mice. a Co-immunostaining of RhoA and α-SMA in the mouse aorta. Nuclei were counterstained with DAPI. **b** Hemodynamics in control and RhoA cKO mice. BP: blood pressure. n=5, each group. **c** External appearance of the extracted aorta with the heart and kidneys. **d** Ultrasonographic observation of the abdominal aorta, which is outlined by yellow dotted lines. **e** Summary graph of the abdominal aortic diameter. n=5, each group. **f** H-E staining of the aorta. **g** Verhoeff van Gieson staining of the aorta. **h** qPCR analysis of genes related to aortic elasticity. *Gapdh* mRNA was used as a control. n=3, each

group. **i** qPCR analysis of collagen type I α1 (*Col1a1*). *Gapdh* mRNA was used as a control. n=9, each group. **j** Contents of total, cross-linked and non-cross-linked collagens in the aorta, which were analyzed by the hydroxyproline assay. Scale bars: 50 μm (**a**, **f**, **g**) and 1 mm (**d**). In (**b**, **e**, **h**, **i**, **j**), the data between the two groups were analyzed by *t*-test.

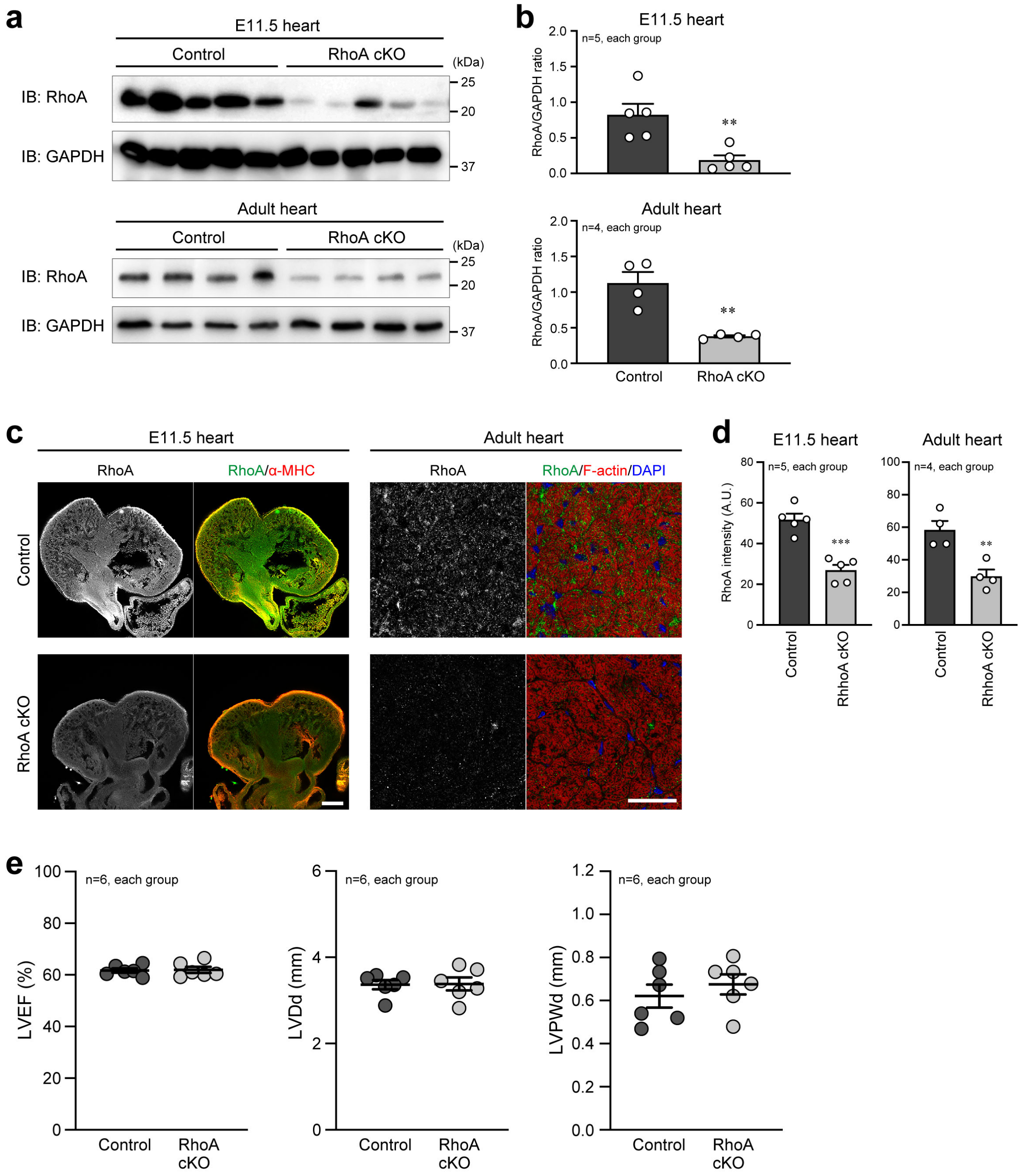

Supplementary Fig. 2 Myocardial RhoA expression and cardiac function in VSMC-specific RhoA cKO mice. a Western blotting for RhoA in the heart samples from embryonic day 11.5 (E11.5) and adult (12 weeks old) mice. GAPDH served as the loading control. **b** Summary graphs of the RhoA/GAPDH ratio examined in (**a**). n=5, each group in E11.5, and n=4, each group in Adult. **c** Immunostaining of RhoA and α-myosin heavy chain (α-MHC) in the mouse heart. F-actin and nuclei were visualized with phalloidin and DAPI, respectively. Scale bars: 200 μm in the left panel and 20 μm in the right panel. **d** Summary graphs of the RhoA intensity in (**c**). A.U.: arbitrary unit. n=5, each group in E11.5, and n=4, each group in Adult. **e** Echocardiographic measurements of parameters related to cardiac function in the adult mice. LVEF: left ventricular ejection fraction, LVDd: left ventricular diastolic diameter, LVPWd: left ventricular posterior wall thickness in diastole. n=6, each group. In (**b**, **d**, **e**), the data between the two groups were analyzed by *t*-test. ** p<0.01 and *** p<0.001vs. Control.

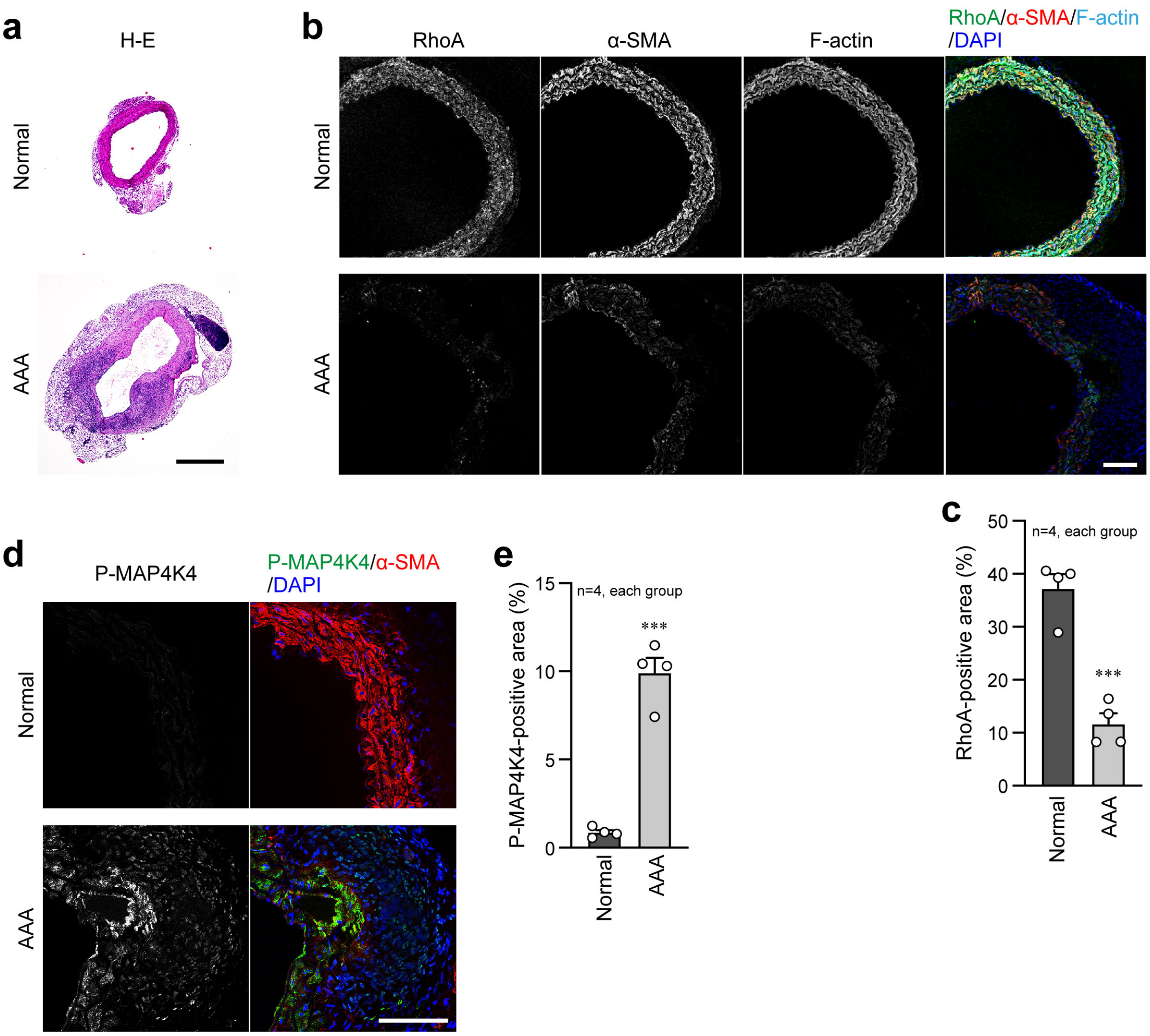

Supplementary Fig. 3 AAA formation in control mice treated with AngII and BAPN. a H-E staining of normal and AAA lesions in the control mice aorta after treatment with AngII and BAPN for 4 weeks. **b**, **d** Co-immunostaining of RhoA and α-SMA (**b**) or P-MAP4K4 and α-SMA (**d**) in the mouse aorta after the treatment. F-actin and nuclei were visualized with phalloidin and DAPI, respectively. **c**, **e** Summary graph of the percentage of RhoA- or P-MAP4K4-positive area. n=4, each group. Scale bars: 500 μm (**a**) and 100 μm (**b**, **d**). The data between the two groups were analyzed by *t*-test. *** $p<0.001$ vs. Normal.

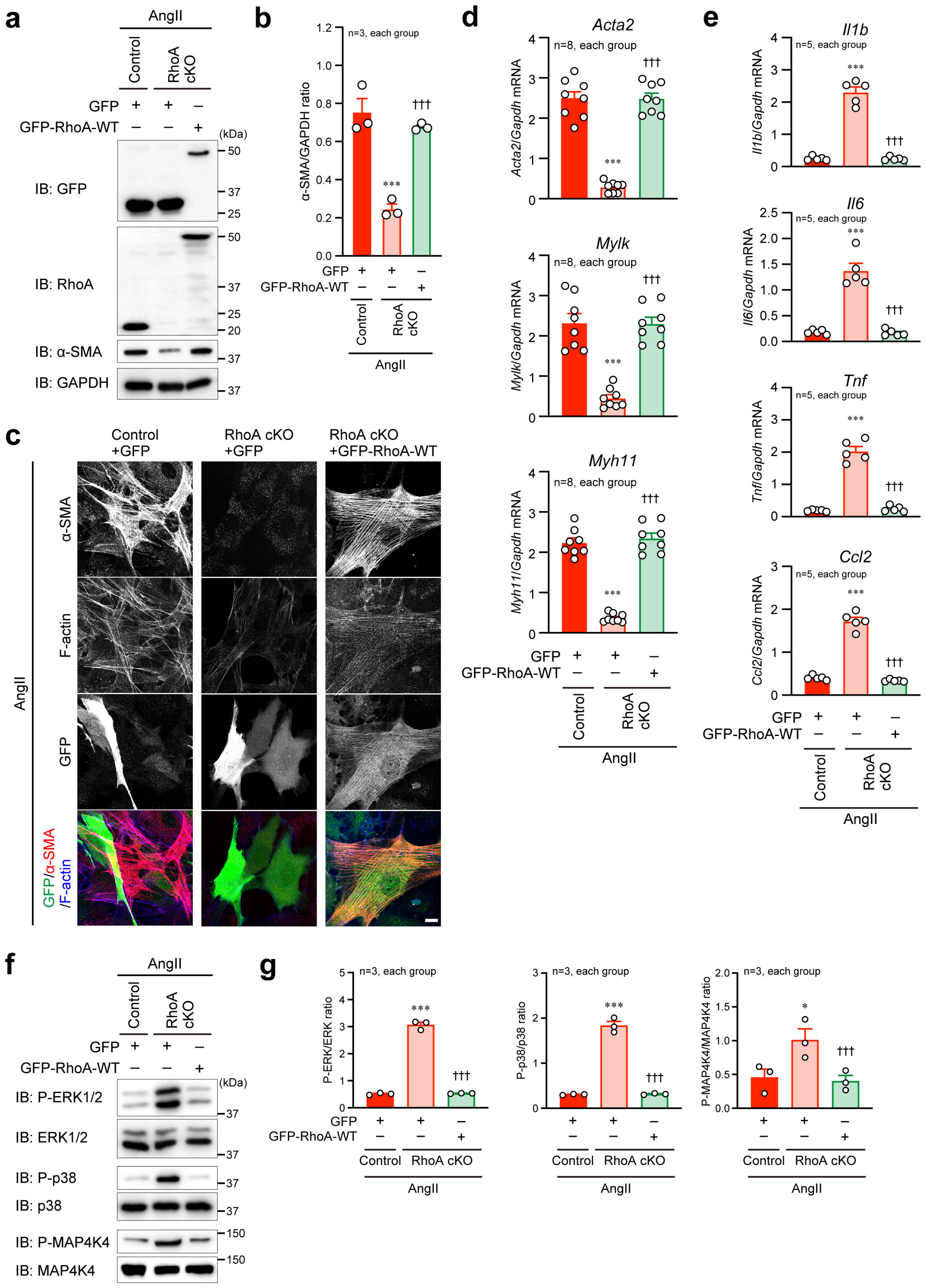

Supplementary Fig. 4 Remedy experiments by re-expression of RhoA in RhoA cKO aortic VSMCs. a After transfection of GFP or GFP-RhoA-WT expression plasmid, VSMCs were stimulated

with AngII for 24 h. The cell extracts were immunoblotted with the indicated antibodies. **b** Summary graph of the α-SMA/GAPDH ratio in (**a**). n=3, each group. **c** Immunofluorescence images of aortic VSMCs stained with the α-SMA antibody after treatment with AngII for 24 h. F-actin was visualized with phalloidin. Scale bar: 20 μm. **d**, **e** qPCR analyses of genes related to VSMC contractile phenotype (**d**) and inflammation (**e**). Aortic VSMCs were treated with AngII for 24 h. n=8, each group. **f** Western blotting with the indicated antibodies using aortic VSMCs after treatment with AngII for 24 h. GAPDH served as the loading control. **g** Summary graphs of the phosphorylated/total MAP kinase signaling molecule ratios examined in (**f**). n=3, each group. In (**b**, **d**, **e**, **g**), one-way ANOVA was applied to compare the data between groups. * $p<0.05$ and *** $p<0.001$ vs. Control; ††† $p<0.001$ vs. GFP-transfected RhoA cKO VSMCs.

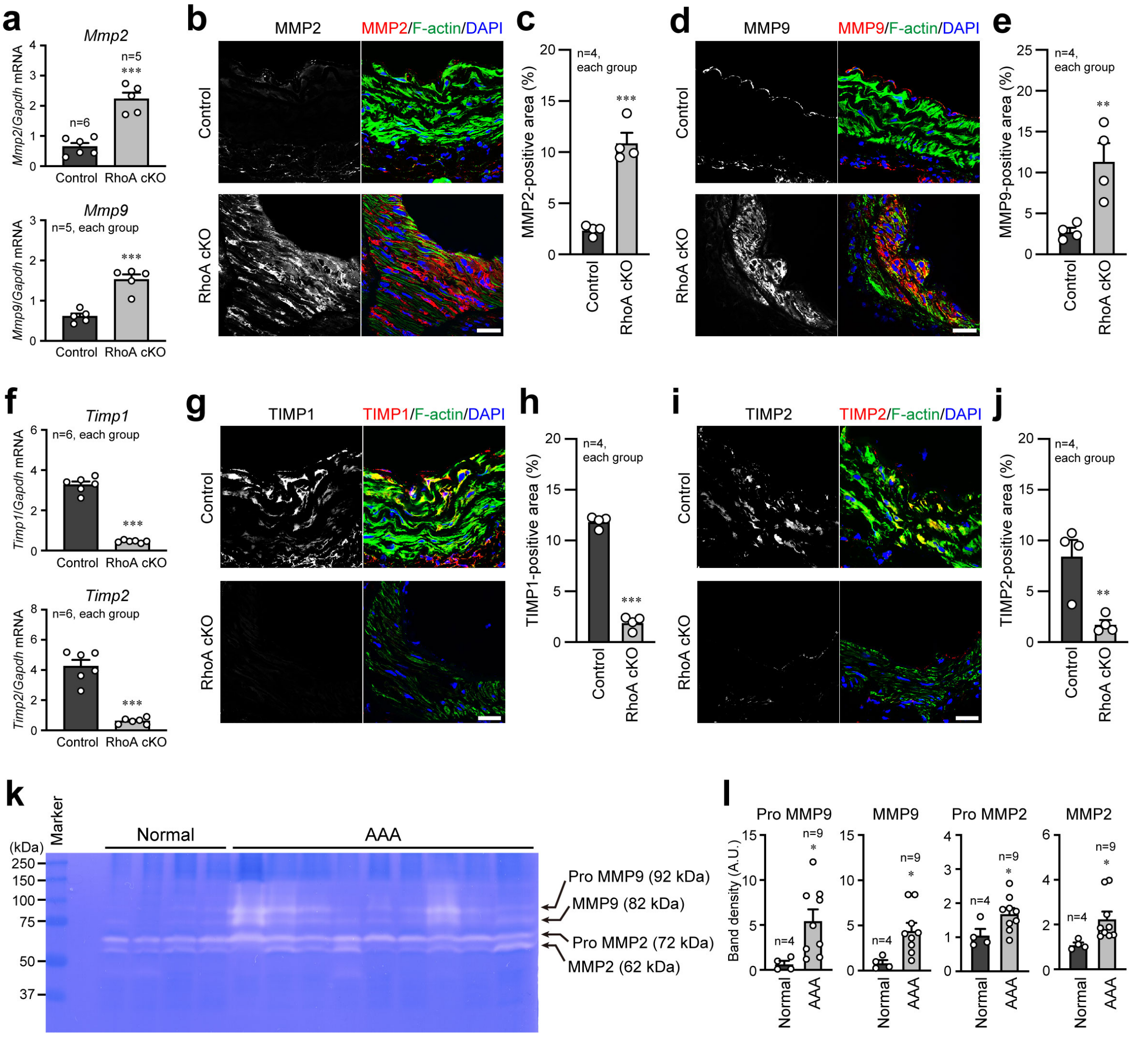

Supplementary Fig. 5 Expression of molecules related to extracellular matrix degradation of the aorta. a, **f** qPCR for MMP or TIMP mRNAs in aortic VSMCs after treatment with AngII for 24 h. n=5–6, each group. **b**, **d**, **g**, **i** Immunofluorescence images of MMPs or TIMPs in the control and RhoA cKO mouse aortas after the 4-week treatment with AngII+BAPN. F-actin and nuclei were visualized with phalloidin and DAPI, respectively. **c**, **e**, **h**, **j** Summary graph of the percentage of the positive area detected by antibody for each MMP or TIMP. n=4, each group. **k** Zymography using human aortic tissue samples. **l**. Summary graphs of band densities observed in (**k**). A.U.: arbitrary unit. n=4 in Normal and n=9 in AAA. Scale bars: 30 μm (**b**, **d**, **g**, **i**). In (**a**, **c**, **e**, **f**, **h**, **j**, **l**), the data between the two groups were analyzed by *t*-test. * p<0.05, ** p<0.01 and *** p<0.001 vs. Control or Normal.

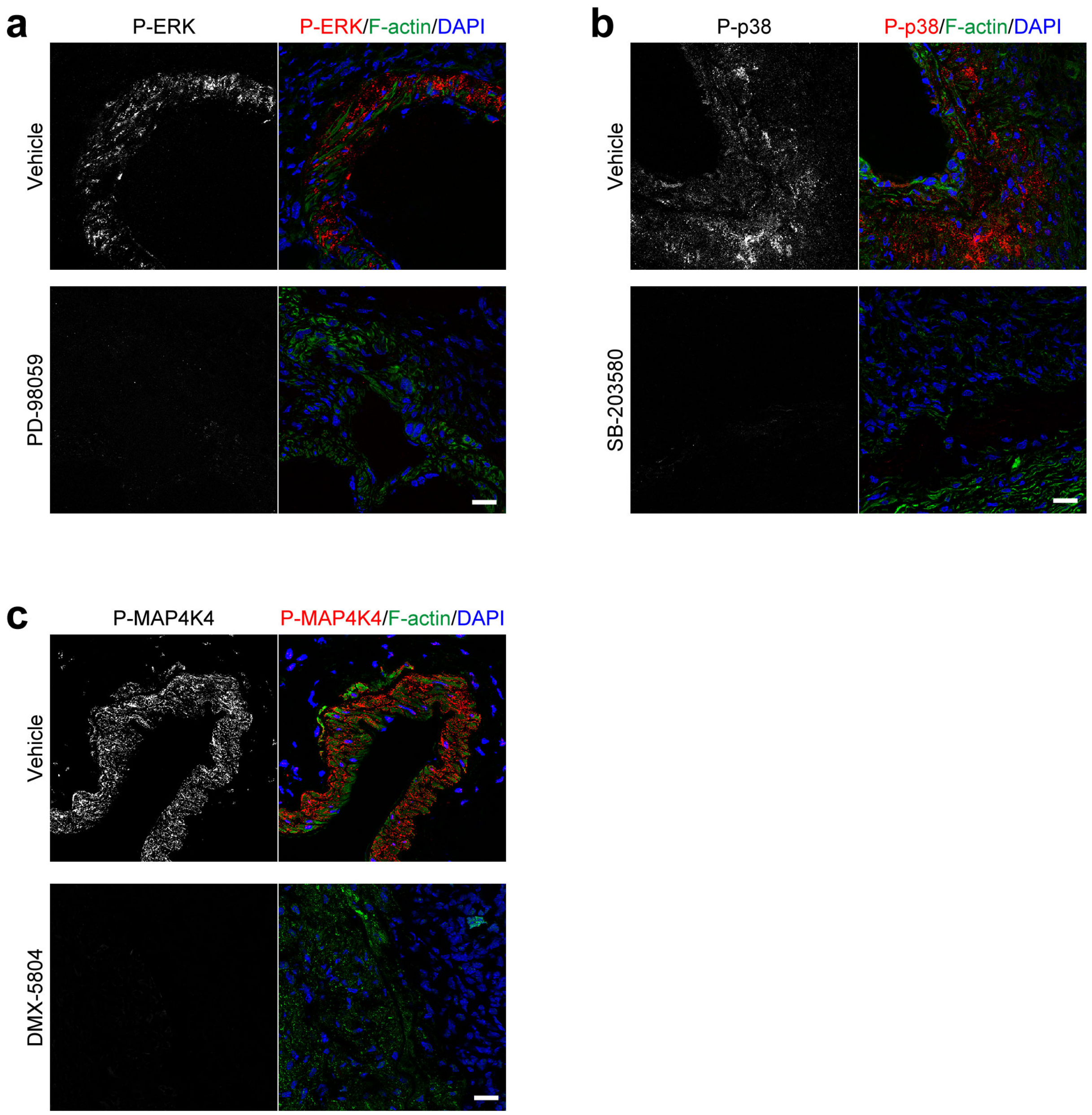

Supplementary Fig. 6 Specificity of the antibodies used in immunofluorescence microscopy to detect each phosphorylated MAP kinase signaling molecule. After RhoA cKO mice were treated with AngII and BAPN for 4 weeks, vehicle (0.5% DMSO) or the inhibitor for ERK (PD-98059, **a**), p38 (SB-203580, **b**) or MAP4K4 (DMX-5804, **c**) was intravenously injected into the mice. At 30 min after the injection, the aorta was extracted and stained with the indicated antibodies. F-actin and nuclei were visualized with phalloidin and DAPI, respectively. Scale bars: 20 μm.

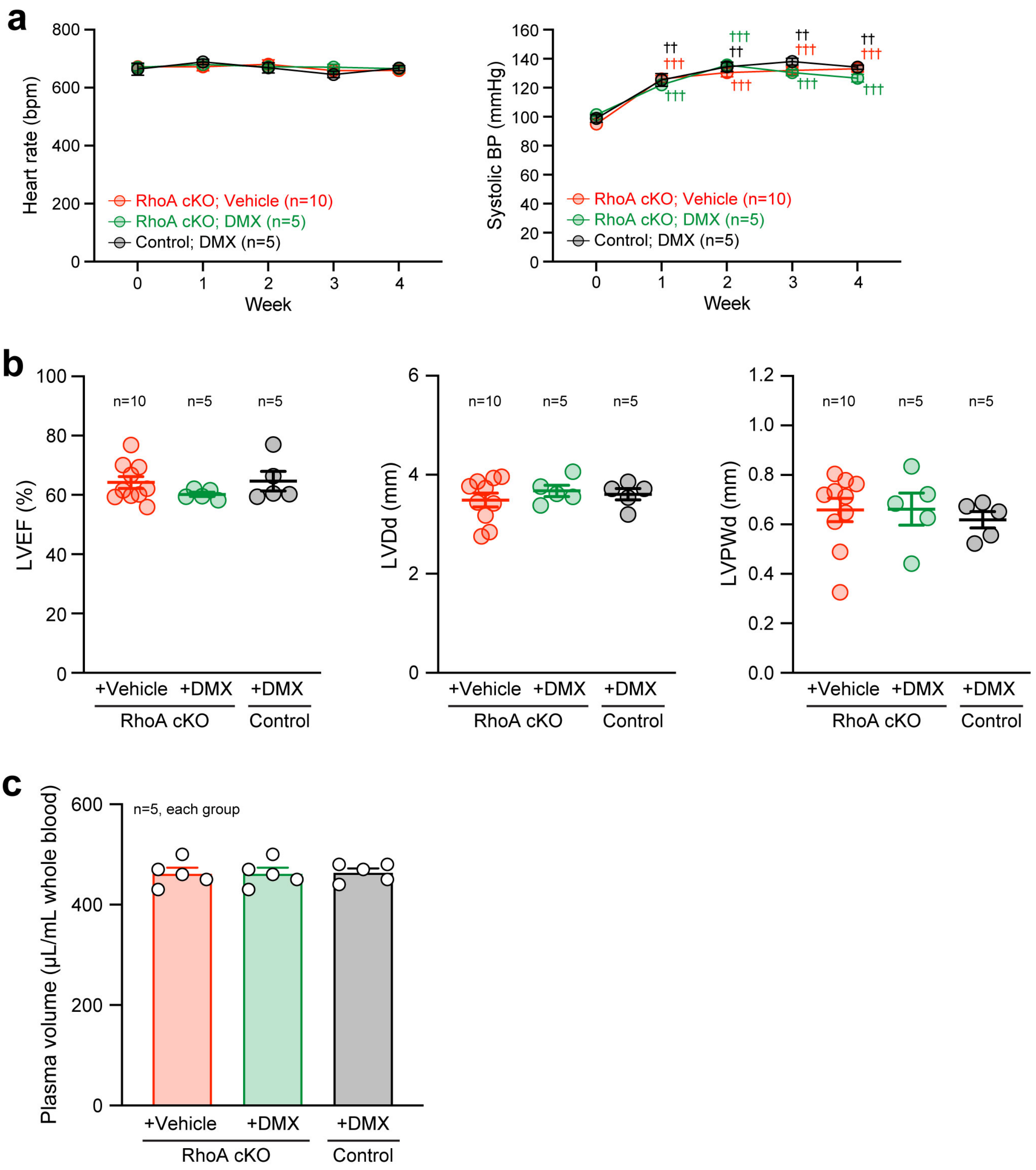

Supplementary Fig. 7 Hemodynamics and cardiac functions after DMX-5804 treatment in addition to AngII and BAPN infusion. **a** Changes in heart rate and systolic BP before (Week 0) and after treatment with AngII+BAPN in the presence of vehicle or DMX-5804. n=5–10, each group. **b** Echocardiographic measurement of parameters related to cardiac function at the end of treatment. LVEF: left ventricular ejection fraction, LVDd: left ventricular diastolic diameter, LVPWd: left ventricular posterior wall thickness in diastole. n=5–10, each group. **c** Plasma volume in 1 mL of whole blood was measured at the end of treatment. n=5, each group. Two-way (**a**) or one-way (**b**, **c**) ANOVA was applied to compare the data between groups. †† p<0.01 and ††† p<0.001 vs. Week 0.

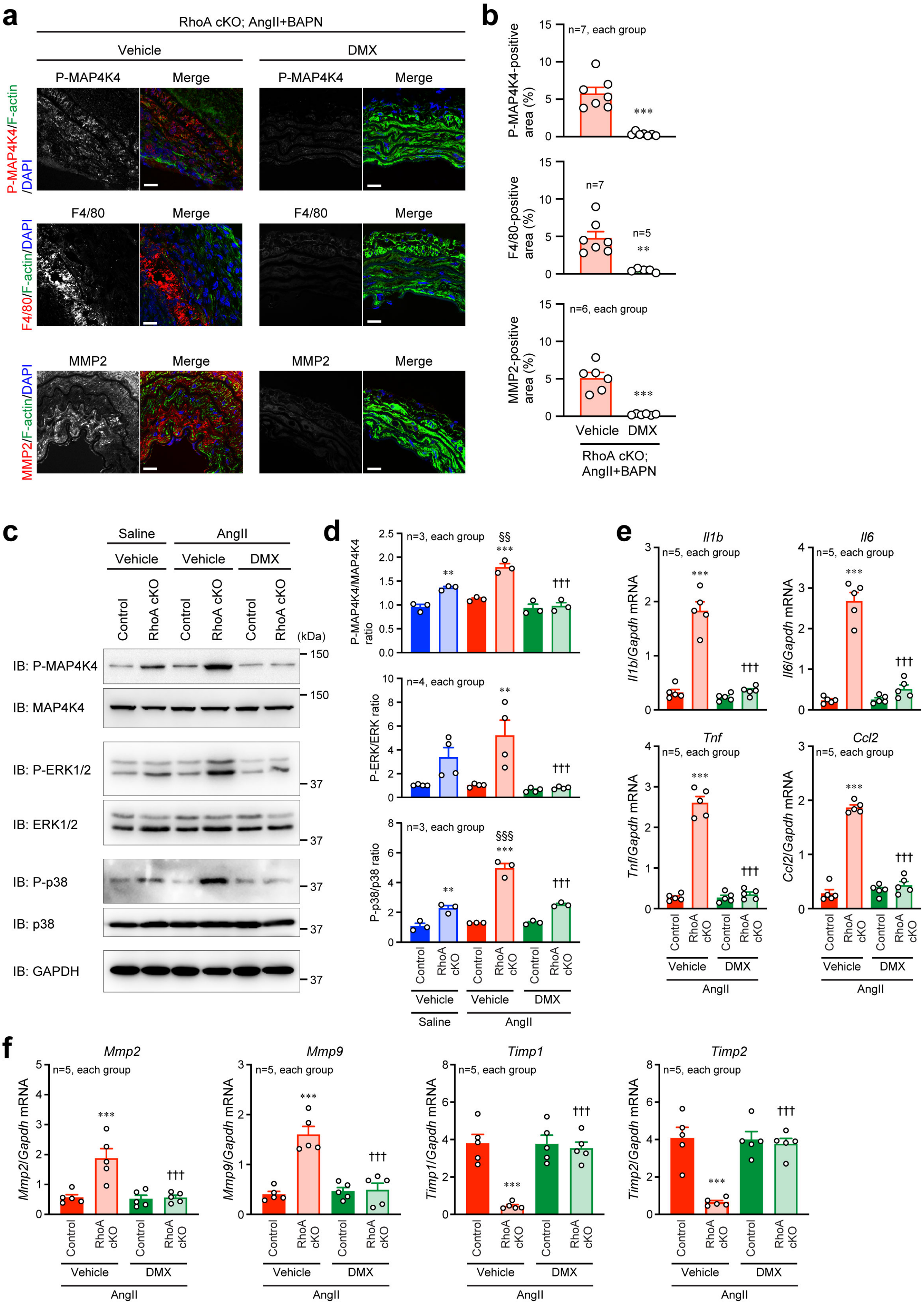

Supplementary Fig. 8 Rescue of RhoA knockout-mediated excessive inflammatory response and MAP kinase signaling activation by DMX-5804 treatment. a Immunofluorescence images of

the control and RhoA cKO mouse aortas stained with the indicated antibodies. The aorta was extracted after the 4-week treatment with AngII+BAPN in the presence of vehicle or DMX-5804. F-actin and nuclei were visualized with phalloidin and DAPI, respectively. Scale bars: 20 μm. **b** Summary graphs of the percentage of the positive area detected by each antibody in the aorta. n=5–7, each group. **c** Western blotting with the indicated antibodies using cultured aortic VSMCs after treatment with saline or AngII in the presence of vehicle or DMX-5804 for 24 h. GAPDH served as the loading control. **d** Summary graphs of the phosphorylated/total protein ratios in each group. n=3–4, each group. **e**, **f** Graphs of qPCR results for inflammatory cytokines, MMPs and TIMPs in aortic VSMCs treated with AngII in the presence of vehicle or DMX-5804 for 24 h. n=5, each group. In (**b**), the data between the two groups were analyzed by *t*-test. One-way (**d**, **e**, **f**) ANOVA was applied to compare the data between groups. ** $p<0.01$ and *** $p<0.001$ vs. Vehicle or Control; ††† $p<0.001$ vs. AngII treatment in the presence of vehicle; §§ $p<0.01$ and §§§ $p<0.001$ vs. Saline.

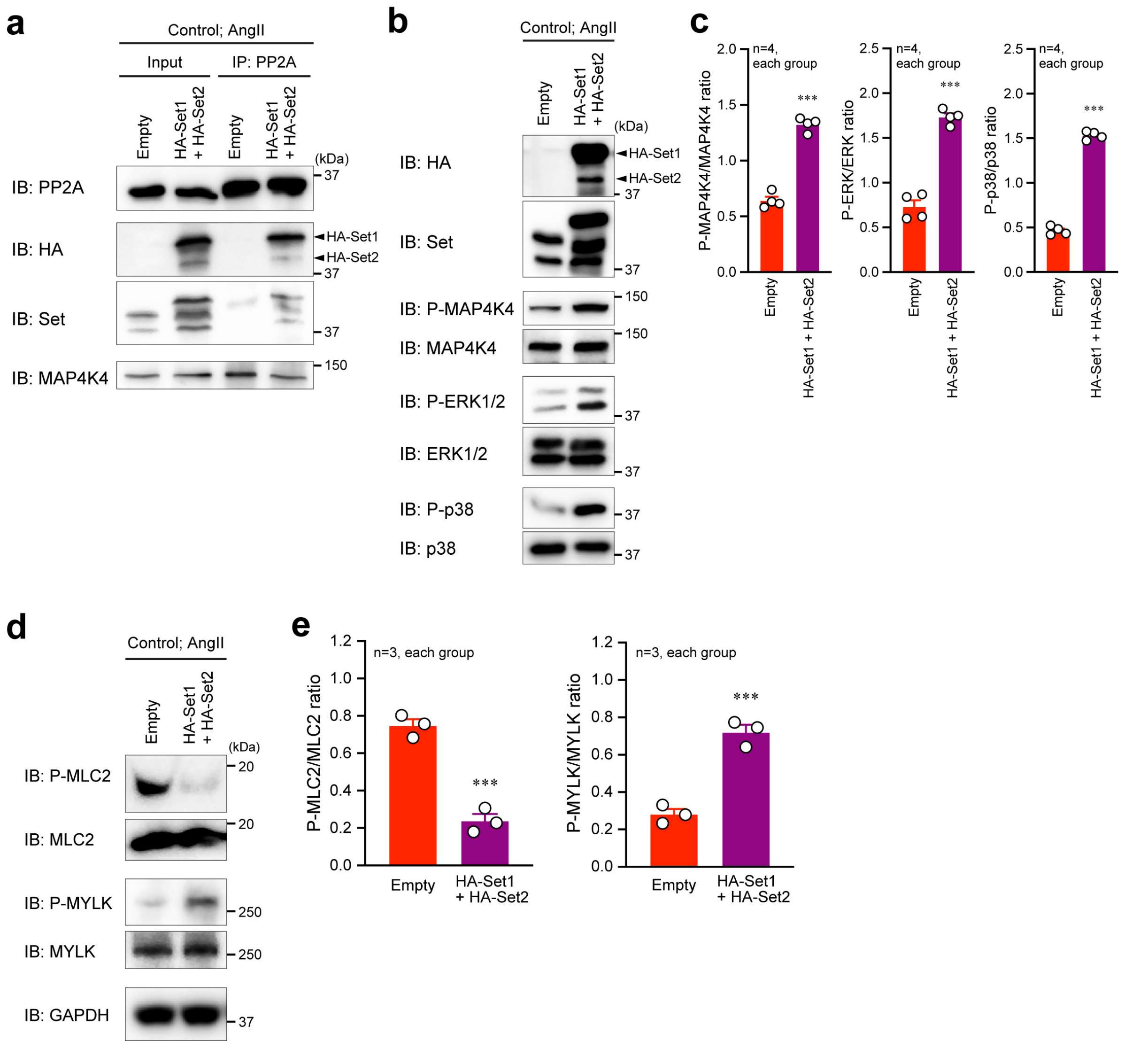

Supplementary Fig. 9 Effect of Set overexpression on aortic VSMCs isolated from control mice. a After control VSMCs transfected with both HA-tagged Set1 and Set2 expression plasmids or an empty plasmid were stimulated with AngII for 24 h, cell extracts were immunoprecipitated with the anti-PP2A antibody, followed by western blotting with the indicated antibodies. **b, d** Cell extracts of control VSMCs treated as described in (**a**) were immunoblotted with the indicated antibodies. **c, e** Summary graphs of the phosphorylated/total MAP kinase signaling molecule ratios (**c**) and the P-MLC2/MLC2 and P-MYLK/MYLK ratios (**e**). n=4 (**c**) and n=3 (**e**), each group. The data were analyzed by *t*-test. *** $p<0.001$ vs. Empty.

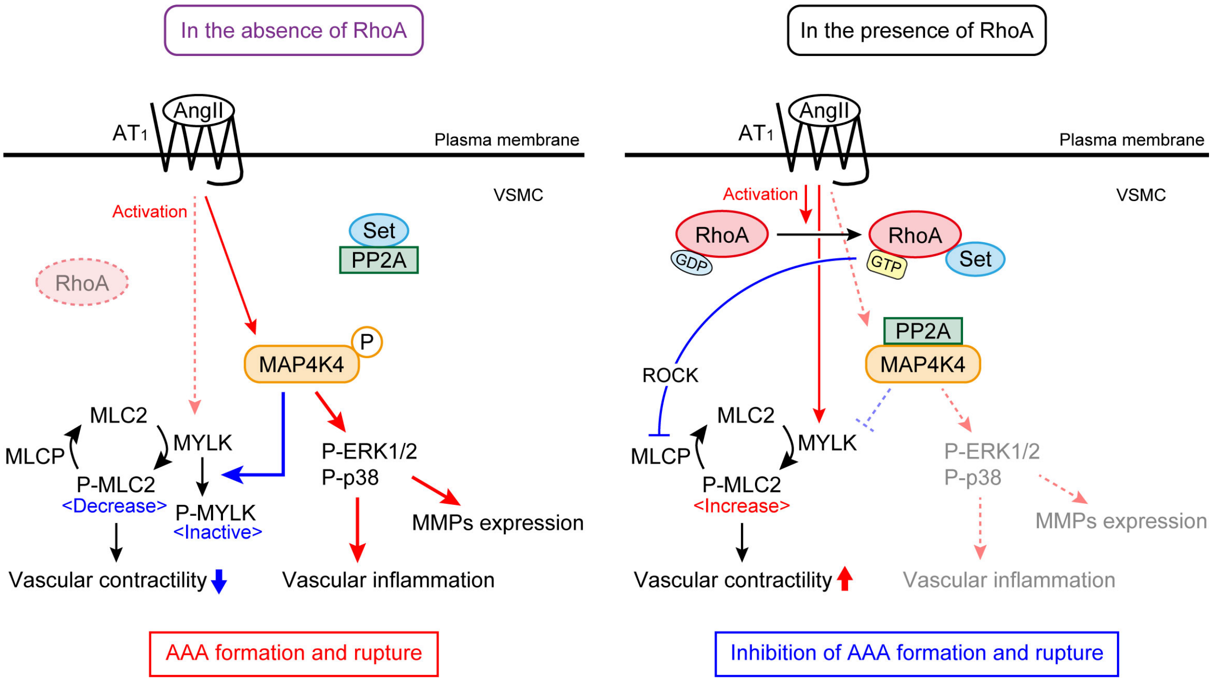

Supplementary Fig. 10 Schematic representation of the protective effect of RhoA on AAA formation. Signal transduction in aortic VSMCs and subsequent feature of the aorta are illustrated in the absence (Left panel) and presence (Right panel) of RhoA in VSMCs.

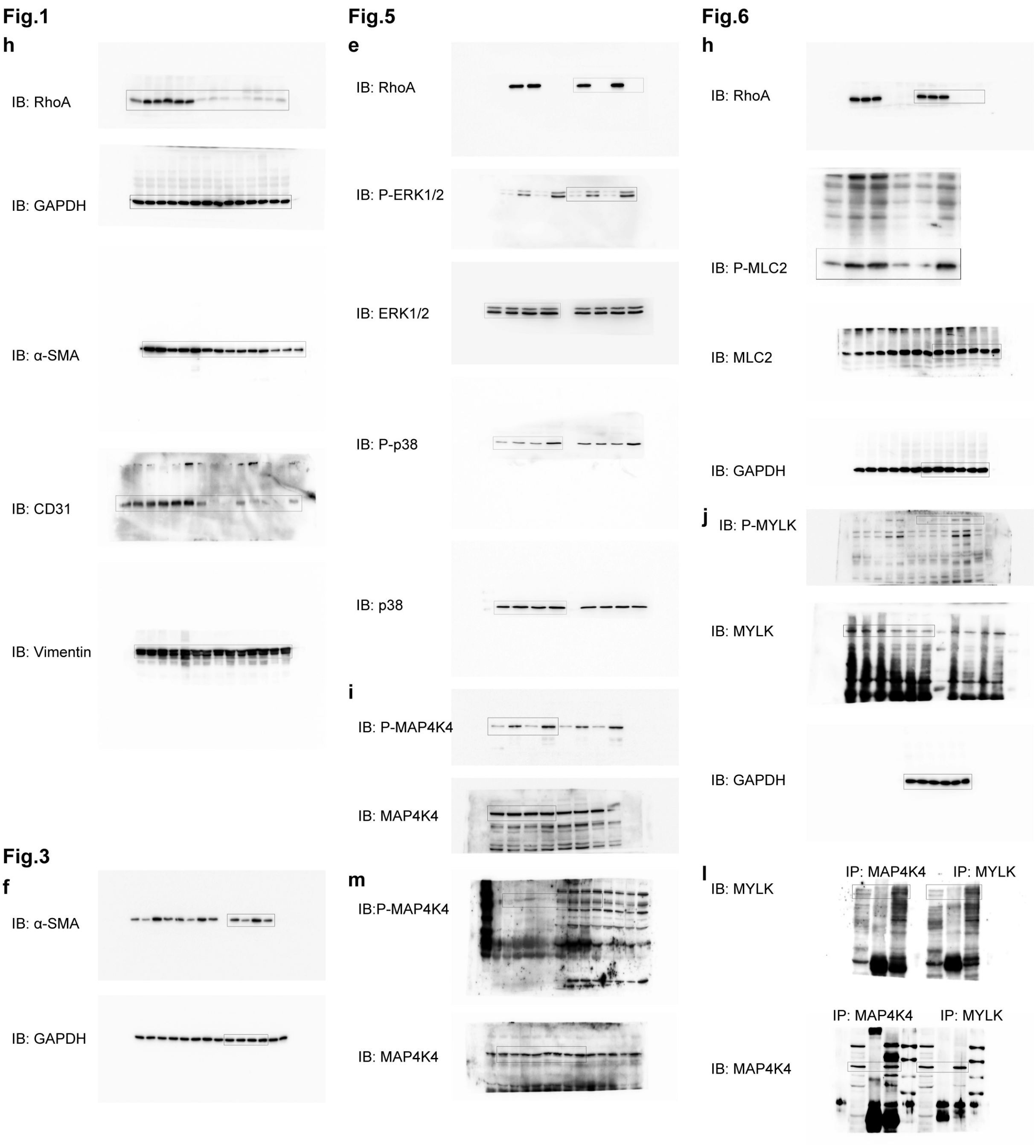

Supplementary Fig. 11 Uncropped western blot images used in Fig. 1, 3, 5 and 6. Rectangles indicate the area shown in each figure panel.

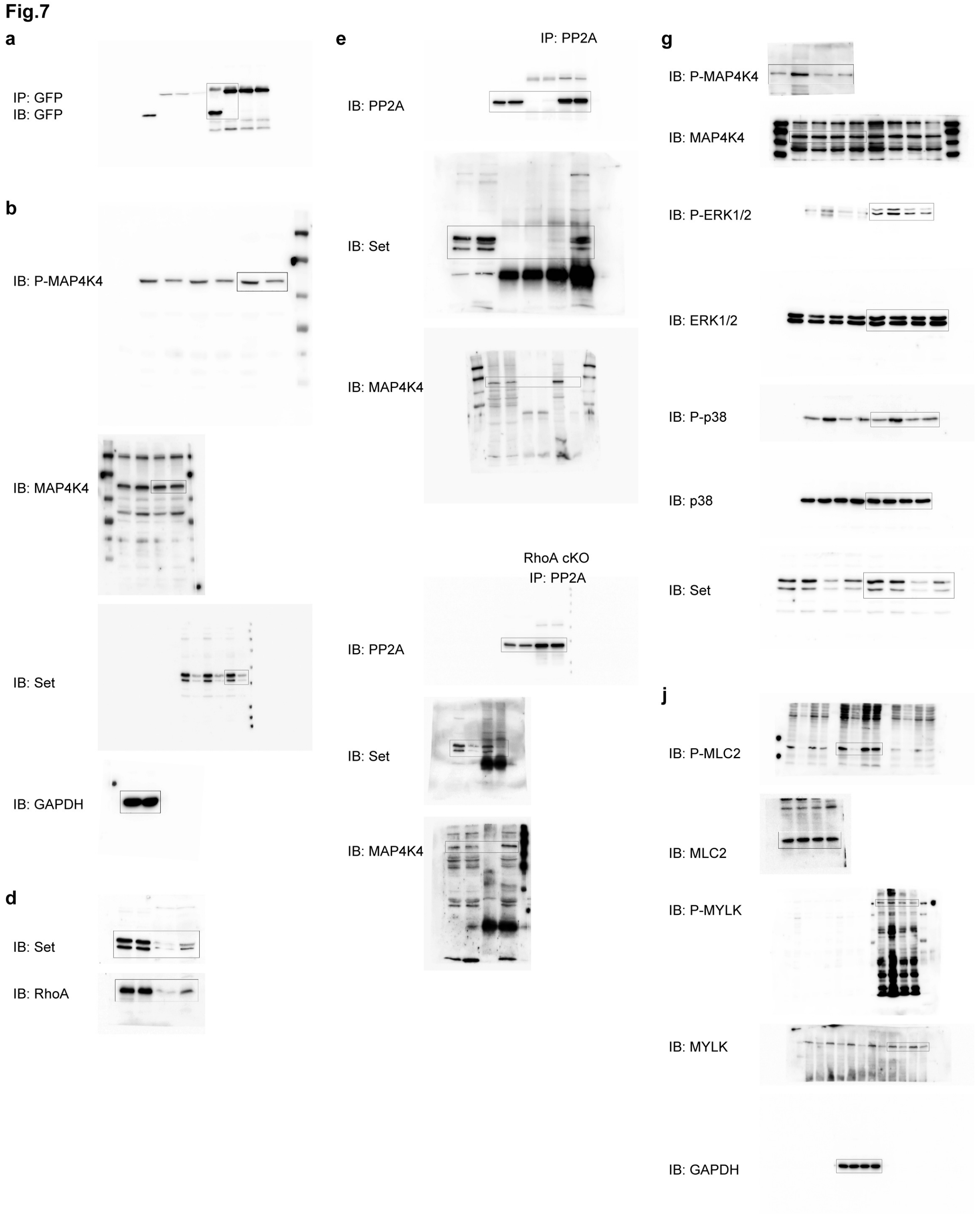

Supplementary Fig. 12 Uncropped western blot images used in Fig. 7. Rectangles indicate the area shown in each figure panel.

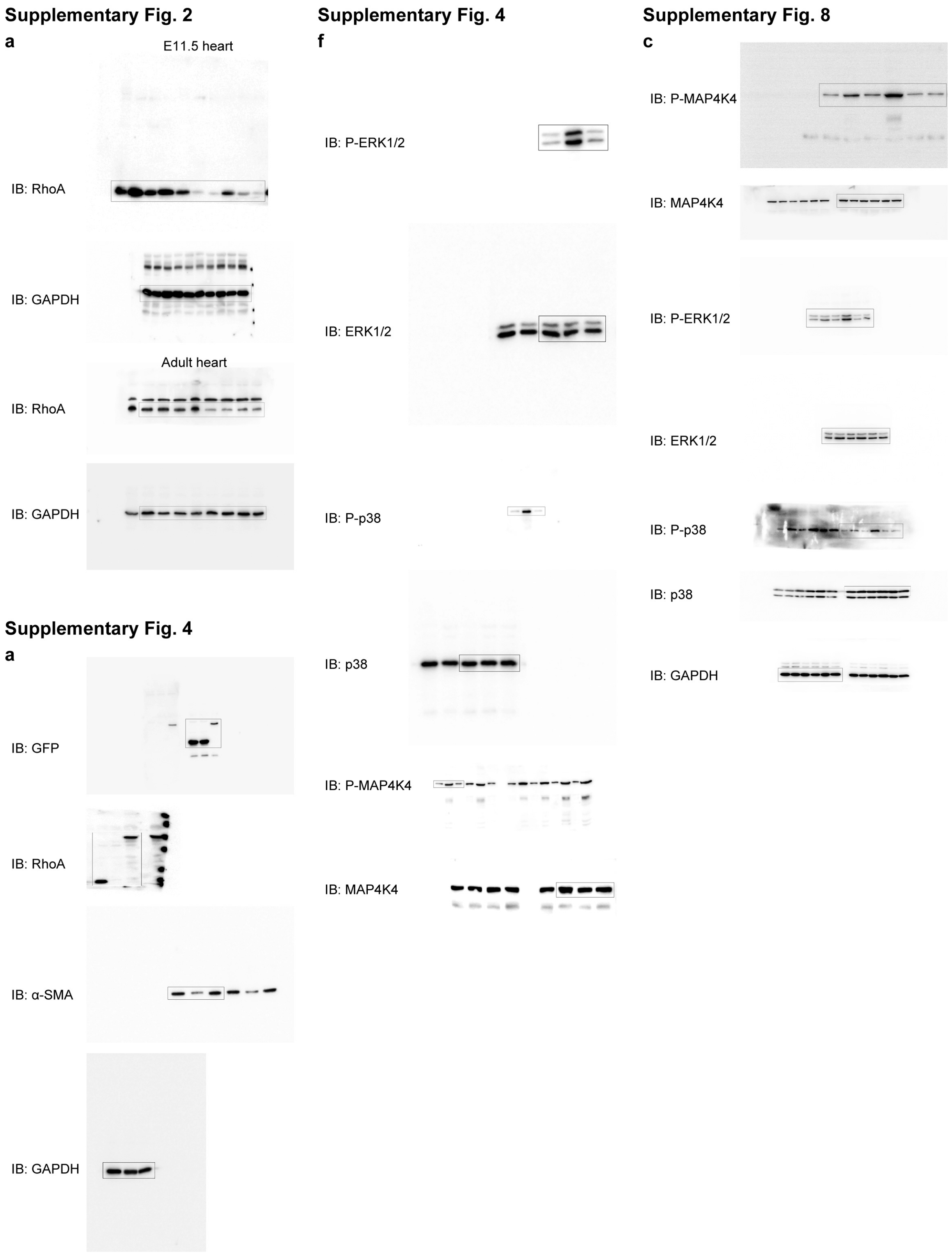

Supplementary Fig. 13 Uncropped western blot images used in Supplementary Fig. 2, 4 and 8. Rectangles indicate the area shown in each figure panel.

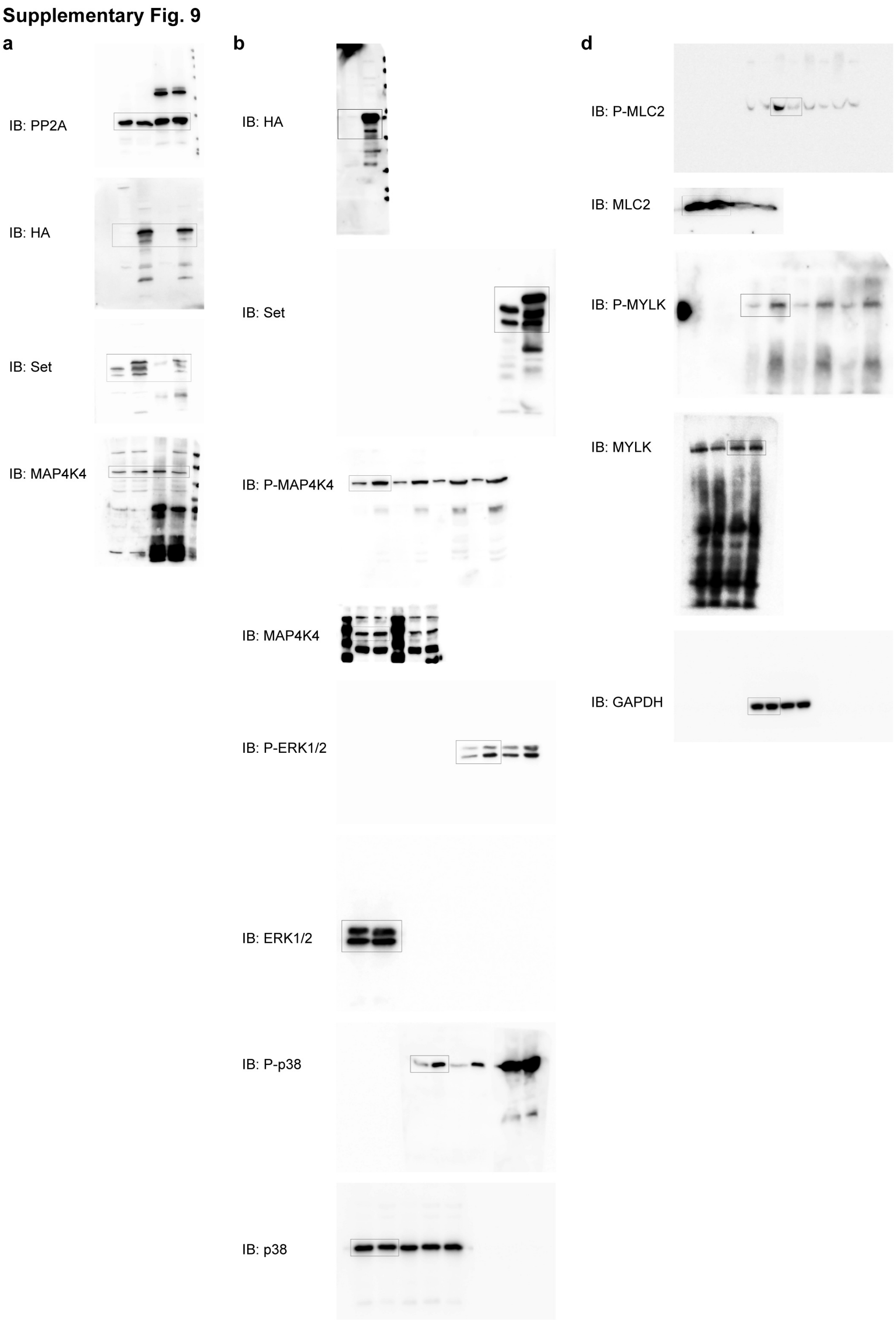

Supplementary Fig. 14 Uncropped western blot images used in Supplementary Fig. 9. Rectangles indicate the area shown in each figure panel.
