# Supplementary material for: Vascular smooth muscle RhoA counteracts abdominal aortic aneurysm formation by modulating MAP4K4 activity
Source: Commun Biol. 2022 Oct 7;5:1071. doi: 10.1038/s42003-022-04042-z (PMC9546906; doi:10.1038/s42003-022-04042-z)
Supplement: Supplementary file 3 — Description of Additional Supplementary Files [file 42003_2022_4042_MOESM3_ESM.pdf]

Description of Additional Supplementary Files

File name: Supplementary Data 1
Description: List of proteins identified by mass spectrometry.

File name: Supplementary Data 2
Description: The source data behind the graphs in the paper.
