# Supplementary material for: Vascular smooth muscle RhoA counteracts abdominal aortic aneurysm formation by modulating MAP4K4 activity
Source: Commun Biol. 2022 Oct 7;5:1071. doi: 10.1038/s42003-022-04042-z (PMC9546906; doi:10.1038/s42003-022-04042-z)
Supplement: Supplementary file 6 — Reporting Summary [file 42003_2022_4042_MOESM6_ESM.pdf]

nature portfolio

Corresponding author(s): Hisakazu Ogita

Last updated by author(s): Sep 17, 2022

Reporting Summary

Nature Portfolio wishes to improve the reproducibility of the work that we publish. This form provides structure for consistency and transparency in reporting. For further information on Nature Portfolio policies, see our Editorial Policies and the Editorial Policy Checklist.

Statistics

For all statistical analyses, confirm that the following items are present in the figure legend, table legend, main text, or Methods section.

n/a	Confirmed	
☐	☒	The exact sample size (*n*) for each experimental group/condition, given as a discrete number and unit of measurement
☐	☒	A statement on whether measurements were taken from distinct samples or whether the same sample was measured repeatedly
☐	☒	The statistical test(s) used AND whether they are one- or two-sided<br>*Only common tests should be described solely by name; describe more complex techniques in the Methods section.*
☒	☐	A description of all covariates tested
☐	☒	A description of any assumptions or corrections, such as tests of normality and adjustment for multiple comparisons
☐	☒	A full description of the statistical parameters including central tendency (e.g. means) or other basic estimates (e.g. regression coefficient) AND variation (e.g. standard deviation) or associated estimates of uncertainty (e.g. confidence intervals)
☐	☒	For null hypothesis testing, the test statistic (e.g. *F*, *t*, *r*) with confidence intervals, effect sizes, degrees of freedom and *P* value noted<br>*Give P values as exact values whenever suitable.*
☒	☐	For Bayesian analysis, information on the choice of priors and Markov chain Monte Carlo settings
☒	☐	For hierarchical and complex designs, identification of the appropriate level for tests and full reporting of outcomes
☒	☐	Estimates of effect sizes (e.g. Cohen's *d*, Pearson's *r*), indicating how they were calculated

Our web collection on statistics for biologists contains articles on many of the points above.

Software and code

Policy information about availability of computer code

Data collection	Multi wire myograph data was collected using LabChart 8 software. Softron data was collected using BP98AWU V1.32. Western blot and real-time PCR data was collected by ( ). LC-MS/MS data was obtained by using PEAKS studio software. Sp8 software was used to get immunofluorescence images. ImageJ software was used for quantification of images and protein band intensity.
Data analysis	Prism 9 software (GraphPad Software, La Jolla, CA, USA). PSOPIA software (National Institute of Biomedical Innovation, Health and Nutrition, Ibaraki, Japan).

For manuscripts utilizing custom algorithms or software that are central to the research but not yet described in published literature, software must be made available to editors and reviewers. We strongly encourage code deposition in a community repository (e.g. GitHub). See the Nature Portfolio guidelines for submitting code & software for further information.

Data

Policy information about availability of data

All manuscripts must include a data availability statement. This statement should provide the following information, where applicable:

- Accession codes, unique identifiers, or web links for publicly available datasets
- A description of any restrictions on data availability
- For clinical datasets or third party data, please ensure that the statement adheres to our policy

N/A

Field-specific reporting

Please select the one below that is the best fit for your research. If you are not sure, read the appropriate sections before making your selection.

☒ Life sciences ☐ Behavioural & social sciences ☐ Ecological, evolutionary & environmental sciences

For a reference copy of the document with all sections, see nature.com/documents/nr-reporting-summary-flat.pdf

Life sciences study design

All studies must disclose on these points even when the disclosure is negative.

Sample size	Total 106 mice (10–14 weeks old male mice. Control n=47; RhoA cKO n=59) were used in this study. 30 clinical samples were used in this study.
Data exclusions	No mice data were excluded. For clinical data, low GAPDH expression samples were excluded from data analyses.
Replication	All whole tissue samples were processed as 2 independent biosample replicates.
Randomization	No randomization was performed
Blinding	No blinding was necessary

Reporting for specific materials, systems and methods

We require information from authors about some types of materials, experimental systems and methods used in many studies. Here, indicate whether each material, system or method listed is relevant to your study. If you are not sure if a list item applies to your research, read the appropriate section before selecting a response.

Materials & experimental systems

n/a	Involved in the study	
☐	☒	Antibodies
☒	☐	Eukaryotic cell lines
☒	☐	Palaeontology and archaeology
☐	☒	Animals and other organisms
☒	☐	Human research participants
☐	☒	Clinical data
☒	☐	Dual use research of concern

Methods

n/a	Involved in the study	
☒	☐	ChIP-seq
☒	☐	Flow cytometry
☒	☐	MRI-based neuroimaging

Antibodies

Antibodies used

RhoA (67B9) Rabbit Monoclonal Cell Signaling Technology 2117 WB (1:1000), IF andIHC (1:200)
RhoB (C-5) Mouse Monoclonal Santa Cruz Biotechnology sc-8084 IF (1:200)
GAPDH (3H12) Mouse Monoclonal Medical & Biological Laboratories M171-3 WB (1:1000)
α-Actin (1A4) Mouse Monoclonal Santa Cruz Biotechnology sc-32251 WB (1:1000), IF (1:200)
α-MHC (MF20) Mouse Monoclonal R&D Systems MAB4470 IF (1:500)
Aggrecan (4F4) Mouse Monoclonal Santa Cruz Biotechnology sc-33695 IF (1:200)
CD31 (SZ31) Rat Monoclonal Dianova DIA-310 IF (1:200)
CD31 Rabbit Polyclonal Abcam ab28364 WB (1:1000)
P-p38 MAPK [Thr180/Tyr182] (D3F9) Rabbit Monoclonal Cell Signaling Technology 4511 WB (1:1000), IF and IHC (1:200)
p38 MAPK (D13E1) Rabbit Monoclonal Cell Signaling Technology 8690 WB (1:1000)
P-p44/42 MAPK [Thr202/Tyr204] (D13.14.4E) Rabbit Monoclonal Cell Signaling Technology 4370 WB (1:1000), IF and IHC (1:200)
p44/42 MAPK (137F5) Rabbit Monoclonal Cell Signaling Technology 4695 WB (1:1000)
P-MAP4K4 Rabbit Polyclonal Bioss bs-5491R WB (1:1000), IF and IHC (1:200)
MAP4K4 Rabbit Polyclonal Proteintech 55247-1-AP WB (1:1000), IP (1:100)
CD68 Rabbit Polyclonal Abcam ab125212 IF (1:200)
F4/80 (C-7) Mouse Monoclonal Santa Cruz Biotechnology sc-377009 IF (1:200)
MMP2 (8B4) Mouse Monoclonal Santa Cruz Biotechnology sc-13595 IF (1:200)
MMP9 (7-11C) Mouse Monoclonal Santa Cruz Biotechnology sc-13520 IF (1:200)
TIMP1 (G-6) Mouse Monoclonal Santa Cruz Biotechnology sc-365905 IF (1:200)
TIMP2 (3A4) Mouse Monoclonal Santa Cruz Biotechnology sc-21735 IF (1:200)
GFP (mFX75) Mouse Monoclonal Wako Pure Chemical Industries 012-22541 WB (1:1000)
GFP (mFX73) Mouse Monoclonal Wako Pure Chemical Industries 012-20461 IP (1:100)
P-MLC2 [Thr18/Ser19] Rabbit Polyclonal Cell Signaling Technology 3674 WB (1:1000)
MLC2 (E-4) Mouse Monoclonal Santa Cruz Biotechnology sc-28329 WB (1:1000)

	Phospho-MYLK [Ser1760] Rabbit Polyclonal Invitrogen 44-1085G WB (1:1000)<br>MYLK (A-8) Mouse Monoclonal Santa Cruz Biotechnology sc-365352 WB (1:1000), IP (1:100)<br>PP2A-Cα/β (1D6) Mouse Monoclonal Santa Cruz Biotechnology sc-80665 WB (1:1000), IP (1:100), IF (1:200)<br>Set Rabbit Polyclonal Novusbio NBP1-33713 WB (1:2000), IF (1:200)<br>HA Rabbit Polyclonal Medical & Biological Laboratories 561 WB (1:1000)<br>Vimentin (D21H3) Rabbit Monoclonal Cell Signaling Technology 5741 WB (1:1000)
Validation	All the used antibodies are commercially available and have been validated by the above mention manufacturers. Validation reports can be found on their websites using the catalogue number indicated above.

Animals and other organisms

Policy information about studies involving animals; ARRIVE guidelines recommended for reporting animal research

Laboratory animals	We used VSMCs specific RhoA cKO mice and for control, C57Bl/6 strain mice , which were purchased from Jackson Laboratory (Bar Harbor, ME, USA).
Wild animals	This study does not involve wild animals.
Field-collected samples	N/A
Ethics oversight	All animal experiments were approved by Shiga University of Medical Science Animal Care and Use Committee (Approval No. 2019-4-5) and were performed in accordance with relevant guidelines and regulations including Animal Research Reporting of In Vivo Experiments (ARRIVE) guidelines.

Note that full information on the approval of the study protocol must also be provided in the manuscript.

Clinical data

Policy information about clinical studies

All manuscripts should comply with the ICMJE guidelines for publication of clinical research and a completed CONSORT checklist must be included with all submissions.

Clinical trial registration	N/A
Study protocol	All protocols using human aorta samples were approved by the Research Ethics Committee at Shiga University of Medical Science. Aortic tissues were obtained from patients with AA after surgery. All patients provided written informed consent for the use of aortic tissues for this study.
Data collection	Abdominal aortic aneurysm specimens collected from patients were immediately stored at -80℃, and the samples stored within three years were used for the analysis in this study.
Outcomes	We hypothesized that the expression of RhoA is reduced at the aneurysm lesions, and assessed the hypothesis by qPCR, western blotting and immunohistochemistry.
